# Prevalência de tromboembolismo pulmonar incidental em pacientes oncológicos: análise retrospectiva em grande centro

**DOI:** 10.1590/1677-5449.002117

**Published:** 2017

**Authors:** Renata Mota Carneiro, Bonno van Bellen, Pablo Rydz Pinheiro Santana, Antônio Carlos Portugal Gomes

**Affiliations:** 1 Hospital Beneficência Portuguesa de São Paulo – BP, Serviço de Cirurgia Vascular, São Paulo, SP, Brasil.; 2 Hospital Beneficência Portuguesa de São Paulo – BP, Serviço de Radiologia, São Paulo, SP, Brasil.

**Keywords:** tromboembolismo pulmonar, câncer, incidental

## Abstract

**Contexto:**

Devido à maior aplicação de exames de imagem rotineiros, especialmente nos pacientes com neoplasia para controle da doença, vem aumentando o diagnóstico de tromboembolismo pulmonar (TEP) incidental, importante fator de morbimortalidade associado.

**Objetivo:**

Identificar os casos de TEP incidental em pacientes oncológicos submetidos a tomografia computadorizada (TC) de tórax, correlacionando aspectos clínicos e fatores de risco associados.

**Métodos:**

Estudo retrospectivo de todos os episódios de TEP ocorridos de janeiro de 2013 a junho de 2016, com seleção dos pacientes oncológicos e divisão deles em dois grupos: com suspeita clínica e sem suspeita clínica (incidentais) de embolia pulmonar.

**Resultados:**

Foram avaliados 468 pacientes com TEP no período citado. Destes, 23,1% eram oncológicos, entre os quais 44,4% apresentaram achado incidental de embolia pulmonar na TC de tórax. Não houve diferença estatística entre os grupos para sexo, idade e tabagismo. Quanto à procedência, 58,3% dos pacientes sem suspeita clínica eram de origem ambulatorial e 41,7% com suspeita de TEP vinham do pronto-socorro (p < 0,001). As neoplasias mais prevalentes foram de pulmão (17,6%), intestino (15,7%) e mama (13,0%). Aqueles com achado incidental apresentaram significativamente mais metástases, sem diferença entre os grupos para realização de quimioterapia, radioterapia ou cirurgia recente. Quanto aos sintomas apresentados, 41,9% daqueles sem suspeita clínica tinham queixas sugestivas de TEP quando realizaram o exame.

**Conclusão:**

TEP incidental é frequente em pacientes oncológicos, especialmente naqueles provenientes de seguimento ambulatorial e em estágios avançados da doença. Sintomas sugestivos de TEP estavam presentes em pacientes sem suspeita clínica ao realizarem a TC de tórax.

## INTRODUÇÃO

O tromboembolismo pulmonar (TEP) possui quadro clínico multifacetado e natureza muitas vezes inespecífica, variando da forma assintomática a casos fatais, o que torna sua incidência populacional provavelmente subestimada. Estudos sobre sua epidemiologia no Brasil são escassos e baseados em dados de autópsias, com estimativa de prevalência de 3,9 a 16,6%^1^^-^ 7. Esses resultados são semelhantes aos encontrados nos Estados Unidos, onde a prevalência varia de 3,4 a 14,8%, com estimativa anual de 600.000 novos casos e 50.000 a 100.000 óbitos^8^^,^ 9. Um recente estudo brasileiro realizado entre 1989 e 2010 identificou 92.999 óbitos causados por TEP como causa básica no país^10^.

Diversos estudos randomizados com dados de autópsias em hospitais mostraram que a taxa de TEP sem suspeita clínica antes do óbito é ainda muito elevada, variando de 67 a 91%, apesar da melhoria dos recursos diagnósticos e do aumento dos conhecimentos sobre a doença. Essa taxa elevada de subdiagnóstico é provavelmente um reflexo marcante da alta mortalidade do TEP quando seu diagnóstico não é estabelecido e, portanto, ele não é tratado^5^^,^ 11 ^-^ 13, podendo chegar a 30% nesses casos^14^.

A associação entre a doença oncológica e o tromboembolismo venoso (TEV) é bem conhecida, com risco quatro a sete vezes superior de esses pacientes desenvolverem um evento trombótico quando comparados à população em geral^15^. O TEV é considerado a segunda causa mais frequente de óbito em pacientes com câncer, além de ser responsável por maiores riscos de complicações hemorrágicas durante o tratamento anticoagulante e de trombose venosa recorrente do que em pacientes sem neoplasia^16^. Uma metanálise de estudos com necropsias mostrou que o TEP foi causa de óbito em 8 a 35% dos casos e contribuiu para o êxito fatal em pelo menos 45% dos casos. Outro fato alarmante veio de um estudo inglês no qual dos 79.733 óbitos descritos como secundários à neoplasia, na realidade, em 7.500 deles a real causa de morte foram eventos de embolia pulmonar fatal que poderiam ter sido evitados^17^.

Devido à maior aplicação de exames de imagem rotineiros e à qualidade dos tomógrafos mais modernos com múltiplos detectores e, portanto, maior sensibilidade, o TEP incidental tem se tornado um achado relativamente comum, especialmente em pacientes oncológicos, que são frequentemente submetidos a TC para controle evolutivo e terapêutico da doença. Esses casos sem suspeita clínica para realização do exame não são necessariamente assintomáticos, porém, devido à própria doença de base, não foram identificados naquele momento como relacionados a uma embolia pulmonar^18^.

O objetivo deste estudo foi identificar os casos de TEP incidental em pacientes oncológicos submetidos a TC de tórax, correlacionando aspectos clínicos e fatores de risco associados.

## MÉTODOS

Foi realizada uma análise retrospectiva de todos os episódios de TEP ocorridos no Hospital Beneficência Portuguesa de São Paulo, Brasil, de janeiro de 2013 a junho de 2016, com base no livro de registros para controle interno do hospital, no qual são anotados todos os casos positivos de TEP diagnosticados na instituição. A partir dos dados desses pacientes, realizou-se uma consulta ao banco de dados eletrônico do nosso serviço de radiologia. Esse sistema eletrônico contém documentos digitalizados como os pedidos médicos com indicação clínica de TC de tórax; questionário do histórico pessoal do paciente com os seguintes tópicos: profissão, tabagismo (sim, não ou ex-tabagista), sintomas relatados (com as seguintes opções para assinalar: febre, falta de ar, tosse seca, tosse produtiva, dor torácica, emagrecimento e outros), doenças conhecidas, medicamentos em uso, tratamento médico prévio ou atual, cirurgias realizadas e se já foi submetido a exames prévios do tórax; além de anamnese médica feita por um médico radiologista do setor.

Os pacientes oncológicos foram então selecionados e divididos em dois grupos: aqueles que realizaram a TC de tórax com suspeita de TEP e aqueles que realizaram o exame sem suspeita da doença, ou seja, foram submetidos ao exame sob outras hipóteses diagnósticas, com base no pedido médico e/ou história clínica, tendo então o achado incidental de embolia pulmonar.

Foram incluídos todos os pacientes portadores de neoplasia com diagnóstico de TEP, e o critério de exclusão adotado foi desconsiderar as TCs de tórax realizadas para controle evolutivo da embolia pulmonar do mesmo paciente no período estudado. Logo, foi considerado apenas o evento inicial para o diagnóstico.

O protocolo da instituição estabelece que os exames de imagem em pacientes oncológicos sejam realizados com contraste, salvo em pacientes com contraindicações, como alergia ou insuficiência renal, para estudo mais pormenorizado das estruturas vasculares e adjacentes ao tumor. Assim, é possível o diagnóstico de embolia pulmonar em TC de tórax mesmo sem o protocolo específico de TEP.

Analisaram-se os seguintes fatores de risco para doença trombótica, condicionados pelo próprio doente e pela doença oncológica e seu tratamento: idade, sexo, procedência (ambulatorial ou hospitalar), tabagismo, localização do tumor, presença de metástase, tratamento com quimioterapia e/ou radioterapia, cirurgia recente (últimos 30 dias), associação com trombose venosa profunda (TVP), infarto pulmonar e sintomas relatados.

Os dados foram submetidos a uma análise estatística. Inicialmente, todas as variáveis foram analisadas descritivamente. Para as variáveis quantitativas, a análise foi feita através da observação dos valores mínimos e máximos, e do cálculo de médias, desvios padrão e mediana. Para as variáveis qualitativas, calculou-se frequências absolutas e relativas. Para comparar as médias dos dois grupos, foi utilizado o teste *t* de Student e, para testar a homogeneidade entre as proporções, foi utilizado o teste do qui-quadrado ou o teste exato de Fisher. O nível de significância utilizado para os testes foi de 5%.

O comitê institucional local de ética em pesquisa concedeu aprovação (CAAE 60806616.0.0000.5483) para a realização de todas as fases do estudo. Não houve necessidade de termo de consentimento livre e esclarecido por se tratar de análise de dados retrospectivos.

## RESULTADOS

No período de 30 meses analisado, foram diagnosticados 468 casos de TEP. Destes, 108 (23,1%) eram pacientes oncológicos, dos quais 44,4% apresentaram achado incidental de embolia pulmonar na TC de tórax. Já nos pacientes não oncológicos (76,9%), foram identificados apenas 61 casos (16,9%) de TEP sem suspeita clínica, o que representou uma diferença estatística significativa (p < 0,001).

A análise clínica e dos fatores de risco foi realizada apenas para os pacientes oncológicos, divididos em dois grupos conforme explicado previamente – pacientes com suspeita clínica (n = 60) e pacientes sem suspeita clínica/incidentais (n = 48) de TEP. Quanto às variáveis demográficas analisadas (Tabela 1), encontramos mediana de idade semelhante entre os grupos: 63,2 anos com desvio padrão de 12,5 anos para aqueles sem suspeita de TEP e 63,4 anos com desvio padrão de 14,4 anos para aqueles com suspeita de TEP (p = 0,925). Observou-se ligeira predominância do sexo feminino nos portadores de neoplasia (56,5%), porém sem relevância estatística entre os grupos, representando 50% dos casos incidentais e 61,7% dos casos com suspeita de TEP (p = 0,224).

**Tabela 1:** Variáveis demográficas dos pacientes oncológicos.

Variável	Categoria	Suspeita de TEP		p
		Sem (n = 48)	Com (n = 60)	
Idade		63,17 ± 12,50	63,42 ± 14,40	0,925(1)
Sexo feminino		24 (50,0%)	37 (61,7%)	0,224(2)
Origem	Ambulatório	28 (58,3%)	7 (11,7%)	< 0,001(2)
	Internados (enfermaria)	16 (33,3%)	21 (35,0%)	
	UTI	2 (4,2%)	7 (11,7%)	
	Pronto-socorro	2 (4,2%)	25 (41,7%)	
Tabagista^*^	Não	21 (51,2%)	29 (54,7%)	0,672(3)
	Sim	1 (2,4%)	3 (5,7%)	
	Ex	19 (46,3%)	21 (39,6%)	

* 14 pacientes (12,9%) não informaram hábito sobre fumo.

(1) Nível descritivo de probabilidade do teste *t* de Student;

(2) Nível descritivo de probabilidade do teste do qui-quadrado;

(3) Nível descritivo de probabilidade do teste exato de Fisher.

TEP: tromboembolismo pulmonar; UTI: unidade de tratamento intensivo.

O hábito de fumar também não apresentou diferença significativa. A maioria dos pacientes dos dois grupos se declarou não fumante (51,2% *vs.* 54,7% sem e com suspeita de TEP, respectivamente), enquanto 46,3% eram ex-tabagistas e 2,4% ainda fumavam no grupo com achado incidental (p = 0,672).

Quanto à origem desses pacientes, uma maioria significativa daqueles sem suspeita clínica de TEP era proveniente de seguimento ambulatorial (58,3%), enquanto aqueles que realizaram a TC de tórax com suspeita clínica de TEP vinham do pronto-socorro (41,7%) (p < 0,001).

**Figura 1:**
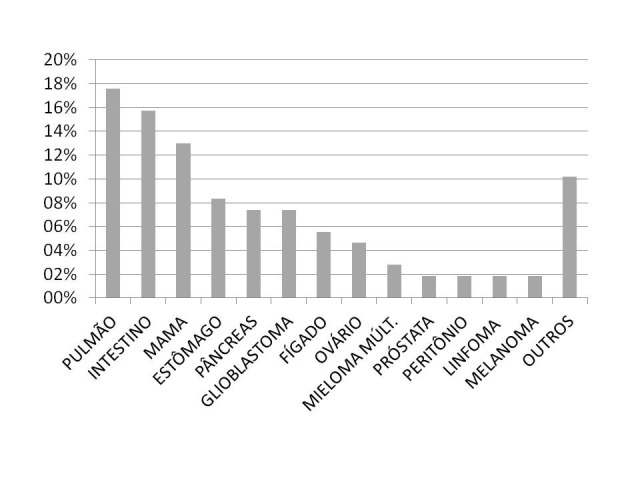
Localização e incidência dos tipos de tumor.

As neoplasias mais prevalentes nos doentes analisados foram: pulmão (17,6%), intestino (15,7%), mama (13,0%), estômago (8,3%), pâncreas e glioblastoma (7,4% cada), conforme relatado na Figura 1.

Foram analisados também alguns fatores de risco relacionados à neoplasia e o tratamento envolvido (Tabela 2). Identificou-se que 66,7% dos pacientes do grupo com achado incidental de TEP eram portadores de metástase, número que diminuiu para 38,3% entre aqueles que fizeram a TC de tórax sob suspeita de embolia (p = 0,003). Não houve diferença estatística entre os grupos quanto à realização de quimioterapia, que foi empregada em 60,4% e 53,3% daqueles sem e com suspeita de TEP, respectivamente; de radioterapia, à qual apenas 20,4% dos pacientes foram submetidos; ou de cirurgia recente realizada nos 30 dias anteriores ao diagnóstico da embolia pulmonar, com resultados semelhantes entre aqueles sem e com suspeita de TEP (12,5% *vs.* 11,7%, respectivamente). Observou-se ainda que 11 (10,2%) pacientes oncológicos apresentaram quadro de TVP associada. Todos foram incluídos no grupo com suspeita de TEP, pois, independentemente dos sintomas apresentados, fizeram a TC de tórax sob suspeita de embolia pulmonar.

**Tabela 2:** Fatores de risco associados.

Variável	Suspeita de TEP		p
	Sem (n = 48)	Com (n = 60)	
TVP	0 (0,0%)	11 (18,3%)	0,001(3)
Cirurgia (30 dias)	6 (12,5%)	7 (11,7%)	0,895(2)
Metástase	32 (66,7%)	23 (38,3%)	0,003(2)
Quimioterapia	29 (60,4%)	32 (53,3%)	0,461(2)
Radioterapia	8 (16,7%)	14 (23,3%)	0,393(2)

(1) Nível descritivo de probabilidade do teste *t* de Student;

(2) Nível descritivo de probabilidade do teste qui-quadrado;

(3) Nível descritivo de probabilidade do teste exato de Fisher.

TEP: tromboembolismo pulmonar; TVP: trombose venosa profunda.

Analisaram-se ainda os sintomas referidos pelos pacientes e/ou a história clínica relatada, sendo que a maioria expressiva dos pacientes dos dois grupos tinha alguma queixa registrada. Havia sintomas relatados em 74,4% daqueles com achado incidental, que vale ressaltar que não está atrelado à falta de sintomas, mas sim ao fato de não ser considerado uma possibilidade diagnóstica na solicitação do exame pelo não reconhecimento dos sintomas associados ao TEP, muitas vezes mascarados pela própria doença de base ou por serem realmente assintomáticos. Já 91,1% dos pacientes oncológicos com suspeita de TEP tiveram sintomas relatados (p = 0,026) ao realizarem a TC de tórax.

Entre os sintomas relatados, observou-se diferença estatística para falta de ar (p < 0,001) e tosse seca (p = 0,017), com a maioria dos casos no grupo com suspeita de TEP, como esperado pela fisiopatologia da doença, com 66,1% e 32,1%, respectivamente, contra 18,6% e 11,6%, respectivamente, no grupo com achado incidental. A segunda queixa mais frequente no grupo com suspeita de TEP foi a dor torácica, presente em 35,7% dos casos, porém sem relevância (p = 0,061) quando comparado ao grupo com achado incidental. No grupo sem suspeita clínica de TEP, a queixa mais relatada foi o emagrecimento, com 48,8% dos casos (p = 0,061), e a queixa com significância estatística foi a dor abdominal (14,0%), justamente por ser um sintoma inespecífico, sendo que nos seis pacientes relatados representava a localização tumoral ou *status* pós-cirúrgico, todos eles no grupo com achado incidental de TEP. Não houve diferença para presença de febre (p = 0,752) e tosse produtiva (p = 1,000) entre os grupos (Tabela 3).

**Tabela 3:** Sintomas relatados na realização da tomografia computadorizada de tórax.

Variável	Categoria	Suspeita de TEP		p
		Sem (n = 48)	Com (n = 60)	
Sintomas^*^		32 (74,4%)	51 (91,1%)	0,026(2)
	Febre	4 (9,3%)	7 (12,5%)	0,752(3)
	Falta de ar	8 (18,6%)	37 (66,1%)	< 0,001(2)
	Tosse seca	5 (11,6%)	18 (32,1%)	0,017(2)
	Tosse produtiva	4 (9,3%)	6 (10,7%)	1,000(3)
	Dor torácica	8 (18,6%)	20 (35,7%)	0,061(2)
	Emagrecimento	21 (48,8%)	17 (30,4%)	0,061(2)
	Dor abdominal	6 (14,0%)	0 (0,0%)	0,005(3)
Infarto pulmonar		7 (14,6%)	14 (23,3%)	0,254(2)

* Nove pacientes (8,3%) não tinham relato de presença ou não de sintomas.

(1) Nível descritivo de probabilidade do teste *t* de Student;

(2) Nível descritivo de probabilidade do teste qui-quadrado;

(3) Nível descritivo de probabilidade do teste exato de Fisher.

TEP: tromboembolismo pulmonar.

Analisamos também a presença de infarto pulmonar, que ocorre quando as artérias brônquicas não são capazes de promover circulação colateral para o segmento pulmonar não perfundido, com possibilidade de dor torácica com característica de angina de peito nos casos graves, em que a sobrecarga aguda do ventrículo direito pode provocar isquemia miocárdica secundária pelo efeito compressivo^19^. Não houve diferença estatística entre os dois grupos, apesar de o infarto pulmonar estar mais presente no grupo com suspeita de TEP, com 23,3% *vs.* 14,6% entre aqueles sem suspeita (p = 0,254).

Considerando todos os pacientes oncológicos (n = 108), a maioria (76,9%) registrou alguma queixa ao ser submetido a TC de tórax, por ordem de frequência: falta de ar (41,7%), emagrecimento (35,2%) e dor torácica (26,0%). Observou-se ainda que 18 pacientes (41,9%) do grupo com achado incidental apresentavam sintomas sugestivos de TEP, como tosse, falta de ar e dor torácica na realização da TC de tórax.

## DISCUSSÃO

O TEV é altamente prevalente e importante fator de morbimortalidade, principalmente na forma de TEP, uma vez que pacientes com câncer e evento trombótico têm menor sobrevida do que aqueles sem trombose associada^20^. Neste estudo, TEP foi um achado radiológico incidental em quase metade dos pacientes com neoplasia, especialmente naqueles oriundos de seguimento ambulatorial e com presença de metástase. Isso se deve a um estado de hipercoagulabilidade inerente à doença metastática, que confere um risco aumentado em até 20 vezes na comparação com aqueles com doença local^21^, e à maior realização de exames de imagem para *status* da doença nesse subgrupo, o que aumenta a chance de achado incidental.

Um estudo espanhol prospectivo observacional realizado entre 2006 e 2009 apresentou dados semelhantes aos nossos: dos 138 pacientes oncológicos com TEP analisados, 45% tiveram achado incidental, sendo 87% provenientes de seguimento ambulatorial e 85% com metástase^22^. Em outro estudo retrospectivo realizado entre 2009 e 2013 que incluiu apenas pacientes oncológicos ambulatoriais, o achado de TEP incidental foi ainda mais significativo: 69,4% dos casos, sendo que 66,1% tinham doença metastática^23^.

Encontramos uma média de idade de 63 anos similar entre os dois grupos e uma discreta predominância do sexo feminino no geral (56,5%). Autores como Exter et al.^24^ não encontraram diferença significativa em relação a sexo ou idade, enquanto Font et al.^25^ identificaram que os pacientes com achado incidental eram 3 anos mais velhos.

Observamos que mais da metade dos pacientes de ambos os grupos realizava tratamento quimioterápico, o que confere um risco aumentado de evento trombótico em duas a seis vezes^21^. Apenas 20,4% dos pacientes tinham sido submetidos a radioterapia, que não foi considerada um fator de risco independente para TEV^26^. O tabagismo é outro fator de risco que confere risco aumentado para evento trombótico^15^, sendo que 46,8% dos pacientes oncológicos com TEP analisados se declararam fumantes ou ex-fumantes.

Outro fator de risco analisado foi a realização de procedimento cirúrgico recente previamente ao diagnóstico do TEP: aproximadamente 12% dos pacientes de ambos os grupos tinham sido submetidos a alguma cirurgia 30 dias antes da TC de tórax. Desses, 46,2% não tinham suspeita clínica de embolia pulmonar e realizaram o exame sob outras indicações, como complicações e/ou seguimento pós-operatório ou da própria doença de base.

Dados da literatura mostram que a incidência de TEV em pacientes oncológicos submetidos a cirurgia é estimada em 37%, sendo que uma grande proporção dos casos ocorre no período pós-alta e o risco pode persistir por até 6 semanas^21^. Além disso, pacientes com achado incidental são menos propensos à hospitalização antes do diagnóstico do TEP^20^, dado coincidente com o encontrado neste estudo, em que apenas 37,5% daqueles sem suspeita clínica eram provenientes do ambiente hospitalar ao realizarem a TC de tórax.

Vale ressaltar que a Associação Americana de Oncologia Clínica determina que a maioria dos pacientes com câncer ativo hospitalizados deve receber tromboprofilaxia durante a internação. Nos casos cirúrgicos, ela é indicada antes de procedimentos de grande porte, como cirurgias abdominais e pélvicas correspondentes, e por pelo menos 7 a 10 dias após o procedimento, podendo ser estendida por até 4 semanas para pacientes com risco elevado^27^.

A localização tumoral também é um fator de risco relevante para o evento trombótico, que apresenta risco aumentado para as neoplasias gastrointestinais, pulmonares, ginecológicas, cerebrais, pancreáticas e os linfomas^21^^,^ 23. No presente estudo, os eventos de TEP foram mais frequentes nos tumores de pulmão (17,6%), intestino (15,7%), mama (13%), estômago (8,3%), pâncreas e glioblastoma (7,4% cada), o que representa não só o sítio tumoral como fator predisponente, mas também o perfil dos doentes seguidos na instituição. Apesar de a neoplasia de mama não estar entre as mais predisponentes para TEV, ela ocupa o terceiro lugar encontrado, o que reflete sua alta incidência populacional, além do frequente tratamento com tamoxifeno, um exemplo de hormonioterapia que se associa a maior risco de evento trombótico^28^.

Conforme elucidado nos resultados, vimos que 74,4% dos pacientes com achado incidental tinham alguma queixa ao realizarem a TC de tórax. O emagrecimento foi citado por 21 deles (48,8%), o que, embora sem relevância estatística, reforça a origem ambulatorial desse grupo por ser um sintoma comum da própria neoplasia e o exame de imagem realizado para seguimento terapêutico de rotina.

Notou-se ainda que uma parcela significativa dos pacientes sem suspeita clínica de TEP (41,9%) tinham sintomas sugestivos de embolia pulmonar, como tosse (36%), falta de ar e dor torácica (32% cada), queixas frequentes e facilmente atribuídas à neoplasia, que dificultam seu reconhecimento no curso da doença ao serem atribuídos a outra causa. Conforme relatado pela Sociedade Internacional de Trombose e Hemostasia, o TEV incidental refere-se a TVP ou TEP que não é clinicamente suspeitado no momento do diagnóstico. Embora o TEP incidental possa ser assintomático, cerca de dois terços dos pacientes afetados relatam ter sintomas consistentes com embolia pulmonar, como fadiga ou falta de ar. No entanto, esses sintomas inespecíficos são frequentemente atribuídos ao câncer ou a efeitos secundários do tratamento. Assim, os médicos devem revisar cuidadosamente a apresentação clínica para determinar se um paciente com TEP incidental apresentou sintomas compatíveis com a doença^29^.

Um estudo francês retrospectivo realizado entre 2005 e 2010 também encontrou dados que corroboram esses achados, demonstrando que 41% dos pacientes com neoplasia e diagnóstico incidental de TEP apresentavam sintomas sugestivos como dispneia (23%), dor torácica (9%) e hemoptise (1%). Já a associação com TVP ocorreu em 8% dos casos^30^. Neste estudo, identificamos uma incidência de 10,2% de TEP em conjunto com TVP, sendo que 36% dos pacientes não tinham queixas respiratórias, porém fizeram a TC de tórax para exclusão diagnóstica.

Devido ao desafio de diagnosticar os casos incidentais em uma população tão frágil, diretrizes internacionais como as do American College of Chest Physicians^31^ recomendam que os pacientes nos quais foi identificado incidentalmente TEP assintomático recebam a mesma anticoagulação inicial e de longo prazo que aqueles com TEP sintomático (grau 2B). Já quanto à tromboprofilaxia de forma ambulatorial, o segmento mais acometido pela embolia pulmonar sem suspeita clínica segundo este estudo, não existe unanimidade para a decisão de profilaxia nesses doentes. De forma geral, deve-se analisar fatores de risco adicionais como evento trombótico prévio, imobilização, doença metastática, terapia hormonal ou quimioterapia, sendo a decisão individualizada para cada paciente, conforme recomendado pela Associação Americana de Oncologia Clínica^27^. Ainda são necessários mais estudos prospectivos para se observar, na prática, os doentes mais beneficiários e as complicações implícitas do uso de anticoagulantes nessa população.

## CONCLUSÃO

O TEP incidental é frequente nos pacientes oncológicos em nosso meio, especialmente naqueles provenientes de seguimento ambulatorial e em estágio avançado da doença. Além disso, sintomas sugestivos de TEP estavam presentes mesmo em pacientes sem suspeita clínica ao realizarem a TC de tórax. Desta forma, os dados indicam a necessidade de se avaliar rigorosamente os doentes com câncer, e que os profissionais envolvidos no seguimento destes pacientes estejam atentos aos sintomas sugestivos de TEV a fim de realizar seu diagnóstico e tratamento o mais precocemente possível.
