# Supplementary material for: Prevalência de tromboembolismo pulmonar incidental em pacientes oncológicos: análise retrospectiva em grande centro
Source: J Vasc Bras. 2017 Jul-Sep;16(3):232–8. [Article in Portuguese] doi: 10.1590/1677-5449.002117 (PMC5868940; doi:10.1590/1677-5449.002117)
Supplement: Translation

# Translation: Prevalence of incidental pulmonary thromboembolism in cancer patients: retrospective analysis at a large center

**Authors:** Renata Mota Carneiro, Bonno van Bellen, Pablo Rydz Pinheiro Santana, Antônio Carlos Portugal Gomes

**Affiliations:** 1 Hospital Beneficência Portuguesa de São Paulo – BP, Vascular Surgery Service, São Paulo, SP, Brazil.; 2 Hospital Beneficência Portuguesa de São Paulo – BP, Radiology Service, São Paulo, SP, Brazil.

**Keywords:** Pulmonary thromboembolism, cancer, incidental

## Abstract

**Background:**

Increased use of routine imaging exams has led to higher rates of incidental diagnosis of pulmonary thromboembolism (PTE), especially for management of disease in cancer patients, among whom it is an important factor in associated morbidity and mortality.

**Objective:**

To identify cases of incidental PTE in cancer patients examined with computed tomography (CT) of the thorax, correlating clinical features and associated risk factors.

**Methods:**

This is a retrospective study of all episodes of PTE diagnosed between January 2013 and June 2016, selecting cases involving cancer patients and dividing them into two subsets: those with clinical suspicion and those without clinical suspicion of pulmonary embolism (incidental cases).

**Results:**

At total of 468 patients had PTE during the period investigated and 23.1% of them were cancer patients, 44.4% of whom exhibited pulmonary embolism as an incidental finding of a chest CT. There was no statistical difference between the two subsets in terms of sex, age, or smoking. In terms of patients’ admission status, 58.3% of the patients without clinical suspicion were outpatients and 41.7% of those with suspicion of PTE were admitted via the emergency room (p < 0.001). The most common cancer sites were lung (17.6%), intestine (15.7%), and breast (13.0%). Patients whose PTE were diagnosed incidentally exhibited a significantly greater rate of metastases, while there were no differences between the groups in terms of chemotherapy, radiotherapy, or recent surgery. Analysis of symptoms revealed that 41.9% of patients without clinical suspicion had complaints suggestive of PTE when they underwent the CT examination.

**Conclusions:**

Incidental PTE is common among cancer patients, especially those in outpatients follow-up and in advanced stages of the disease. Patients without clinical suspicion also had symptoms suggestive of PTE when they presented for chest CT.

## INTRODUCTION

Pulmonary thromboembolism (PTE) has multifaceted clinical status and is very often nonspecific in nature, ranging from asymptomatic forms to fatal cases, which means that its incidence in the general population is probably underestimated. There are few studies investigating its epidemiology in Brazil and those that exist are based on autopsy data, with prevalence estimated at 3.9 to 16.6%.^1^^-^ 7 These results are similar to findings from the United States, where reported prevalence rates vary from 3.4 to 14.8%, and it is estimate there are 600,000 new cases and 50,000 to 100,000 deaths annually.^8^^,^ 9 A recent Brazilian study conducted with data from 1989 to 2010 identified 92,999 deaths in the country in which PTE was the basic cause.^10^

Several randomized studies using hospital autopsy data have shown that there are still very high rates of PTE in the absence of clinical suspicion prior to death, varying from 67 to 91%, despite improved diagnostic resources and increased knowledge about the disease. This elevated underdiagnosis rate is probably a reflection of the high mortality from PTE when it is not diagnosed and, therefore, goes untreated,^5^^,^ 11 ^-^ 13 which can be as high as 30% of these cases.^14^

The association between oncological diseases and venous thromboembolism (VTE) is well known, and these patients are at four to seven times higher risk of suffering a thrombotic event when compared to the general population.^15^ Venous thromboembolism is considered the second most common cause of death among cancer patients and is also responsible for higher risk of hemorrhagic complications during anticoagulant treatment and of recurrent venous thrombosis than in patients free from neoplasms.^16^ A meta-analysis of autopsy studies showed that PTE was the cause of death in 8 to 35% of cases and that it contributed to the fatal outcome in at least 45% of cases. Another alarming fact was highlighted in an English study that found that out of 79,733 deaths described as secondary to cancer, in 7,500 cases the real cause of death was a fatal pulmonary embolism event that could have been avoided.^17^

One result of the increased use of routine imaging exams and the higher quality of more modern tomography equipment with multiple detectors and higher sensitivity is that incidental PTE has become a relatively common finding, especially in cancer patients, who are frequently subjected to CT for monitoring of disease progression and treatment. These cases in which the examination is not conducted in the light of a clinical suspicion are not necessarily asymptomatic, but because of the underlying disease, the symptoms have not been identified as related to a pulmonary embolism.^18^

The objectives of this study were to identify cases of incidental PTE among cancer patients examined with chest CT and correlate them with clinical features and associated risk factors.

## METHODS

A retrospective analysis was conducted of all episodes of PTE recorded at the Hospital Beneficência Portuguesa in São Paulo, Brazil, from January 2013 to June 2016, using the hospital’s internal control register, in which all positive PTE cases diagnosed at the institution are recorded. These patient data were then used to search our radiology service’s electronic database. This electronic system stores digitized copies of physicians’ requests, including the clinical indications for chest CT; patient history questionnaires covering the following: profession, smoking (yes, no, or ex-smoker), symptoms reported (with the following response options: fever, shortness of breath, dry coughing, productive coughing, chest pain, wasting, and others), known diseases, medications currently taken, prior or current medical treatment, surgery performed, and whether the patient has previously undergone chest scans; in addition to patient histories taken by a radiology specialist from the department.

Cases involving cancer patients were then selected and classified into two subsets: those who had undergone chest CT with a suspicion of PTE and those who had had the CT scan with no prior suspicion of PTE, i.e. those who had been sent for the examination with some other diagnostic hypotheses, based on the physician’s request and/or clinical history, in whom pulmonary embolism was therefore an incidental finding.

The study included all cancer patients diagnosed with PTE, and the only exclusion criterion was to discard chest CTs conducted to monitor progress of a pulmonary embolism in the same patient during the study period. In other words, only the initial event was considered for analysis.

The institution’s protocol defines that imaging examinations of cancer patients are conducted with contrast, except for patients with contraindications, such as allergies or kidney failure, to enable more detailed study of vascular structures and those adjacent to tumors. It is thus possible to diagnose pulmonary embolism during a chest CT even when a specific protocol for PTE is not being followed.

The following risk factors for thrombotic disease related to the patient, their cancer, and its treatment were analyzed: age, sex, admission status (outpatients or inpatients), smoking, tumor site, presence of metastasis, treatment with chemotherapy and/or radiotherapy, recent surgery (during the previous 30 days), association with deep venous thrombosis (DVT), pulmonary infarction, and symptoms reported.

Data were analyzed statistically. Initially, descriptive statistics were calculated for all variables. For quantitative variables, analysis was based on identification of minimum and maximum values and calculation of means, standard deviations, and medians. For qualitative variables, absolute and relative frequencies were calculated. Student’s *t* test was used to compare means across the two subsets and the chi-square test or Fisher’s exact test was used to test for homogeneity of proportions. The significance level was set at 5% for all tests.

The local institutional research ethics committee granted approval (review certificate 60806616.0.0000.5483) for all phases of the study. Free and informed consent forms were unnecessary because the study was based on analysis of retrospective data.

## RESULTS

During the 30-month period analyzed, a total of 468 cases of PTE were diagnosed. Of these, 108 (23.1%) were cancer patients, 44.4% of whom had a pulmonary embolism detected as an incidental finding of chest CT. Among patients who did not have cancer (76.9%), just 61 cases (16.9%) of PTE were identified in the absence of clinical suspicion, which is a statistically significant difference (p < 0.001).

Analysis of clinical variables and risk factors was restricted to the cancer patients, divided into two subsets, as described above: patients with clinical suspicion (n = 60) and (incidental) patients with no clinical suspicion (n = 48) of PTE. With regard to the demographic variables analyzed (Table 1), we found that median age was similar in both subsets: 63.2 years, with a standard deviation of 12.5 years among those without suspicion of PTE and 63.4 years with a standard deviation of 14.4 years for patients in whom PTE was suspected (p = 0.925). There was a slight predominance of females among the cancer patients (56.5%), but the difference between the subsets was not statistically relevant, in which women accounted for 50% of incidental cases and 61.7% of cases in which PTE had been suspected prior to the CT examination (p = 0.224).

**Table 1:** Demographic variables for the sample of cancer patients.

Variable	Category	PTE suspected		p
		No (n = 48)	Yes (n = 60)	
Age		63.17 ± 12.50	63.42 ± 14.40	0.925(1)
Female		24 (50.0%)	37 (61.7%)	0.224(2)
Admission status	Outpatients	28 (58.3%)	7 (11.7%)	< 0.001(2)
	Wards	16 (33.3%)	21 (35.0%)	
	ICU	2 (4.2%)	7 (11.7%)	
	Emergency room	2 (4.2%)	25 (41.7%)	
Smoker^*^	No	21 (51.2%)	29 (54.7%)	0.672(3)
	Yes	1 (2.4%)	3 (5.7%)	
	Ex-smoker	19 (46.3%)	21 (39.6%)	

* 14 patients (12.9%) did not provide information on smoking.

(1) Descriptive level of probability according to Student’s *t* test;

(2) Descriptive level of probability according to chi-square test;

(3) Descriptive level of probability according to Fisher’s exact test.

PTE: pulmonary thromboembolism; ICU: intensive care unit.

There was also no significant difference with relation to the variable smoking habit. The majority of patients in both subsets stated they were non-smokers (51.2% vs. 54.7% in subsets without and with suspicion of PTE, respectively), while 46.3% were ex-smokers and 2.4% of those in the incidental finding group were active smokers (p = 0.672).

With regard to the admission status of these patients, a significant majority of those without clinical suspicion of PTE were outpatients (58.3%), while among those who had undergone a chest CT with clinical suspicion of PTE, the largest proportion were from the emergency room (41.7%) (p < 0.001).

The most prevalent tumor sites among the patients analyzed were: lung (17.6%), intestine (15.7%), breast (13.0%), stomach (8.3%), and pancreas and glioblastoma (both 7.4%), as illustrated in Figure 1.

**Figure 1:** Site and incidence of types of tumor.

Certain risk factors related to patients’ neoplasms and the treatment involved were also analyzed (Table 2). It was found that 66.7% of the patients in the incidental PTE subset had metastases, while the equivalent proportion was 38.3% among those whose chest CT examinations were conducted with a suspicion of embolism (p = 0.003). There was no statistical difference between the groups in terms of use of chemotherapy, which was employed in 60.4% and 53.3% of patients without and with suspicion of PTE, respectively; in terms of radiotherapy, which was administered to just 20.4% of patients; or in terms of surgery within the 30 days prior to diagnosis of pulmonary embolism, for which figures were similar among those without and with suspicion of PTE (12.5% and 11.7%, respectively). It was also found that 11 (10.2%) cancer patients had concomitant DVT. All of these were allocated to the group with suspicion of PTE, since irrespective of their symptoms they had all undergone CT scans of the thorax with suspicion of pulmonary embolism.

**Table 2:** Risk factors associated.

Variable	PTE suspected		p
	No (n = 48)	Yes (n = 60)	
DVT	0 (0.0%)	11 (18.3%)	0.001(3)
Surgery (within 30 days)	6 (12.5%)	7 (11.7%)	0.895(2)
Metastasis	32 (66.7%)	23 (38.3%)	0.003(2)
Chemotherapy	29 (60.4%)	32 (53.3%)	0.461(2)
Radiotherapy	8 (16.7%)	14 (23.3%)	0.393(2)

(1) Descriptive level of probability according to Student’s *t* test;

(2) Descriptive level of probability according to chi-square test;

(3) Descriptive level of probability according to Fisher’s exact test.

PTE: pulmonary thromboembolism; DVT: deep venous thrombosis.

Symptoms reported by patients and/or their clinical histories were also analyzed, revealing that the great majority of the patients in both subsets had some type of complaint recorded. Symptoms were recorded for 74.4% of the incidental finding patients, highlighting the fact that absence of suspicion was not because of a lack of symptoms, but because PTE was not considered a diagnostic possibility when the examination was requested, because of failure to recognize symptoms associated with PTE, which are very often masked by the underlying disease, or because the patients really were asymptomatic. For 91.1% of the cancer patients in whom PTE was suspected, symptoms were reported when the chest CT was conducted.

Among the symptoms recorded, there were statistical differences for shortness of breath (p < 0.001) and dry coughing (p = 0.017), both with a majority of cases in the group with PTE suspicion, as would be expected in view of the pathophysiology of the disease, with 66.1% and 32.1% respectively, versus 18.6% and 11.6% respectively in the incidental finding group. The second most common symptom in the group with PTE suspicion was chest pain, reported in 35.7% of cases, but the difference in comparison to the incidental finding group was not statistically relevant (p = 0.061). In the subset of patients without clinical suspicion of PTE, the most frequently reported symptom was wasting, in 48.8% of cases (p = 0.061), and abdominal pains (14.0%) exhibited statistical significance, since it is a nonspecific symptom and in all six patients it was related to the tumor site or post-surgical status, all of them in the incidental PTE diagnosis group. There were no differences between groups for fever (p = 0.752) or productive coughing (p = 1.000) (Table 3).

**Table 3:** Symptoms described during computed tomography of the thorax.

Variable	Category	PTE suspected		p
		No (n = 48)	Yes (n = 60)	
Symptoms^*^		32 (74.4%)	51 (91.1%)	0.026(2)
	Fever	4 (9.3%)	7 (12.5%)	0.752(3)
	Shortness of breath	8 (18.6%)	37 (66.1%)	< 0.001(2)
	Dry coughing	5 (11.6%)	18 (32.1%)	0.017(2)
	Productive coughing	4 (9.3%)	6 (10.7%)	1.000(3)
	Chest pain	8 (18.6%)	20 (35.7%)	0.061(2)
	Wasting	21 (48.8%)	17 (30.4%)	0.061(2)
	Abdominal pains	6 (14.0%)	0 (0.0%)	0.005(3)
Pulmonary infarction		7 (14.6%)	14 (23.3%)	0.254(2)

* For nine patients (8.3%), neither presence nor absence of symptoms was reported.

(1) Descriptive level of probability according to Student’s *t* test;

(2) Descriptive level of probability according to chi-square test;

(3) Descriptive level of probability according to Fisher’s exact test.

PTE: pulmonary thromboembolism.

We also analyzed the presence of pulmonary infarction, which occurs when the bronchial arteries are unable to provide collateral circulation to the pulmonary segment that has lost perfusion, and can involve chest pain with characteristics of angina in severe cases, in which an acute overload of the right ventricle can provoke secondary myocardial ischemia due to compressive effects.^19^ There was no statistical difference between the two subsets, although pulmonary infarction was more frequent in the group with suspicion of PTE, in 23.3%, compared to 14.6% of those without suspicion (p = 0.254).

Considering all of the cancer patients together (n = 108), the majority (76.9%) reported at least one complaint when they underwent their chest CT. In descending order of frequency, these symptoms were: shortness of breath (41.7%), wasting (35.2%), and chest pain (26.0%). It is notable that 18 patients (41.9%) in the incidental finding subset had symptoms suggestive of PTE, such as coughing, shortness of breath, and chest pain, when they underwent their CT scans.

## DISCUSSION

Venous thromboembolism is a very prevalent and important factor of morbidity and mortality, primarily in the form of PTE, since cancer patients with thrombotic events have lower survival rates than those who have no associated thromboses.^20^ In this study, PTE was an incidental radiological finding in almost half of the cancer patients and was especially frequent among those in outpatients follow-up and those with metastases. This is because of the hypercoagulable state inherent to metastatic disease, which confers up to 20 times greater risk in comparison with patients with localized disease,^21^ and because of the more frequent use of imaging exams to monitor disease status among this subset of patients, increasing the likelihood of incidental detection.

A prospective observational study conducted in Spain from 2006 to 2009 reported similar data to ours: of 138 cancer patients with PTE analyzed, 45% had incidental findings, 87% of whom were in outpatients follow-up and 85% of whom had metastases.^22^ In another retrospective study, conducted between 2009 and 2013 and which only enrolled cancer outpatients, incidental PTE diagnosis was even more significant at 69.4% of cases, 66.1% of whom had metastatic disease.^23^

We observed a mean age of 63 years, which was similar in both groups and a discrete predominance of female in the overall sample (56.5%). Authors such as Exter et al.^24^ did not find significant differences in relation to sex or age, whereas Font et al.^25^ found that patients with incidental diagnoses were 3 years older.

We observed that more than half of the patients in both groups were given chemotherapy, which confers a two to six times greater risk of thrombotic events.^21^ Just 20.4% of the patients had undergone radiotherapy, which is not considered an independent risk factor for VTE.^26^ Smoking is another risk factor that confers increased risk of thrombotic events,^15^ and 46.8% of the sample of cancer patients with PTE analyzed here stated they were smokers or ex-smokers.

Another risk factor analyzed was surgical procedures performed a short time before diagnosis of PTE: approximately 12% of the patients in both groups had undergone some type of surgery up to 30 days prior to their chest CT scans. Within this subset, 46.2% did not have clinical suspicion of pulmonary embolism and underwent the examination because of other indications, such as complications, postoperative follow-up, and/or for monitoring the underlying disease.

Published data show that the incidence of VTE in cancer patients subjected to surgery can be estimated at 37%, that a large proportion of cases occur after discharge, and that risk can persist for up to 6 weeks.^21^ Additionally, patients with incidental findings are less likely to have been admitted to hospital before PTE is diagnosed,^20^ which was mirrored in this study, where just 37.5% of patients without clinical suspicion were already in hospital when they were sent for the chest CT.

It is worth noting that the American Society of Clinical Oncology states that the majority of patients with active cancer should be given thromboprophylaxis while in hospital. In surgical cases, it is indicated before major procedures, such as abdominal and pelvic surgery, for at least 7 to 10 days after the procedure, and can be continued for up to 4 weeks for patients at high risk.^27^

Tumor site is also a relevant risk factor for thrombotic events, with increased risk associated with gastrointestinal, lung, gynecological, cerebral, pancreatic and lymphomas.^21^^,^ 23 In the present study, PTE events were more frequent in patients with tumors of the lungs (17.6%), intestines (15.7%), breasts (13%), stomach (8.3%) and pancreas (7.4%), and those with glioblastoma (7.4%), which is not merely an indication of the role of tumor site as predisposing factor, but is also a reflection of the profile of patients seen at the institution. While breast cancer is not one of the forms that most predispose to VTE, it was the third most frequent in this sample, which is a reflection of its high incidence in the population, and is also linked to the fact that it is often treated with tamoxifen, a type of hormone therapy that is associated with a higher risk of thrombotic events.^28^

Our results showed that 74.4% of the patients with an incidental PTE finding had some type of complaint when they went for their chest CT. Wasting was mentioned by 21 (48.8%) of them and while this was not statistically relevant, it underscores their outpatients origin, since this is a common symptom of cancer and the imaging exams were requested for routine follow-up of treatment.

It was also observed that a significant proportion of the patients without clinical suspicion of PTE (41.9%) had symptoms suggestive of pulmonary embolism, such as coughing (36%), shortness of breath, and chest pain (both 32%), complaints that are common and easily attributed to cancer, making it less likely that when seen in oncological patients they will be attributed to other causes. According to the International Society on Thrombosis and Haemostasis, incidental VTE is defined as DVT or PTE that was not suspected clinically at the time of diagnosis. Although incidental PTE may be asymptomatic, around two thirds of affected patients reported symptoms consistent with pulmonary embolism, such as tiredness or shortness of breath. However, these nonspecific symptoms are often attributed to cancer or the secondary effects of treatment. Physicians should therefore carefully review clinical presentation to determine whether a patient with incidental PTE exhibited symptoms compatible with the disease.^29^

A retrospective study conducted from 2005 to 2010 in France also reported data supporting these findings, showing that 41% of patients with neoplasms and an incidental PTE diagnosis had suggestive symptoms such as dyspnea (23%), chest pain (9%), and hemoptysis (1%). In 8% of cases there was an association with DVT.^30^ In our study, we identified an incidence of 10.2% of PTE with concomitant DVT, while 36% of these patients did not have respiratory complaints, but had the chest CT for differential diagnosis.

In view of the challenge of diagnosing incidental cases in a very fragile population, international guidelines such as those published by the American College of Chest Physicians recommend that patients in whom asymptomatic PTE is identified incidentally should be given the same initial and long term anticoagulation as those with symptomatic PTE (grade 2B).^31^ However, there is no consensus on thromboprophylaxis for outpatients, which in the present study was the subset in which pulmonary embolism was most often diagnosed in the absence of clinical suspicion. In general, additional risk factors such as prior thrombotic events, immobilization, metastatic disease, hormone therapy, and chemotherapy should be analyzed, and the decision taken on a case-by-case basis, as recommended by the American Society of Clinical Oncology.^27^ Additional prospective studies are still needed in order to observe, in practice, which patients will benefit most and the complications implicit in use of anticoagulants with this population.

## CONCLUSIONS

Incidental PTE is common among the cancer patients seen in our setting, especially those in outpatients follow-up and in advanced stages of the disease. Furthermore, patients without clinical suspicion also had symptoms suggestive of PTE when they presented for chest CT. Therefore, these data reveal a need to rigorously assess cancer patients, and for the professionals involved in managing them to be alert to symptoms suggestive of VTE, in order to achieve diagnosis and treatment as early as possible.
